# Exploring Diet and Nutrient Insufficiencies across Age Groups: Insights from a Population-Based Study of Brazilian Adults

**DOI:** 10.3390/nu16050750

**Published:** 2024-03-05

**Authors:** Mauro Fisberg, Lais Duarte Batista, Agatha Nogueira Previdelli, Gerson Ferrari, Regina Mara Fisberg

**Affiliations:** 1 Centro de Excelência em Nutrição e Dificuldades Alimentares (CENDA), Instituto Pensi, Fundação José Luiz Egydio Setúbal, Hospital Infantil Sabará, São Paulo 01227-200, Brazil; 2 Department of Pediatrics, Federal University of São Paulo, São Paulo 04023-062, Brazil; 3 Department of Nutrition, School of Public Health, University of São Paulo, São Paulo 01246-904, Brazil; laisduarte@usp.br (L.D.B.); rfisberg@usp.br (R.M.F.); 4 Faculty of Health Sciences, Universidad Autónoma de Chile, Providencia 7500912, Chile; agatha.usp@gmail.com (A.N.P.); gersonferrari08@yahoo.com.br (G.F.)

**Keywords:** dietary intake, micronutrients, nutrition surveys, public health

## Abstract

Assessing dietary inadequacies can contribute to understanding the nutritional vulnerabilities of a country. This study aimed to investigate nutrient intakes and micronutrient inadequacies in the Brazilian adult population, with an interest in different age subgroups. We conducted a cross-sectional study with 1812 individuals aged 19 to 65 years from a population-based study with a representative sample of Brazilian adults. Dietary intake was assessed by two 24 h food recalls, and the probabilities of inadequate intake were estimated using the Dietary Reference Intake targets. Adequate macronutrient intake was over 99% for proteins, 84.7% for carbohydrates, and 80.7% for total fats. There was a high probability of inadequacy (above 90%) for vitamins D and E, but vitamin D inadequacy was very similar between the sexes. In contrast, vitamin E was more likely to be inadequately consumed among women. A high probability of inadequacies (above 85%) of calcium and magnesium were found in the population, regardless of age group. Except for iron, the probability of an inadequacy of other minerals increased with age. The results showed a relevant proportion of nutrient inadequacies, with those most at risk being women and older individuals, helping with the better targeting and monitoring of public-health policies that address nutritional problems in the population.

## 1. Introduction

Diet is an important aspect of human health, providing essential nutrients for growth, development, and lifelong health [1]. Suboptimal dietary patterns and inadequate nutrient intakes are associated with several non-communicable diseases (NCDs), such as high blood pressure, obesity, micronutrient deficiencies, and type 2 diabetes, contributing to morbidity and mortality worldwide [2]. In 2017, dietary risk factors accounted for 22% of total adult mortality, highlighting its relevant role as a modifiable risk behavior to promote health [3].

Diet-related health consequences result from a nutritional imbalance in the intake of macronutrients, vitamins, and minerals. High energy, sodium, saturated fat, and refined carbohydrate intakes are usually risk factors for many NCDs [4]. Additionally, deficiencies in calcium, iron, dietary fiber, vitamin D, and other micronutrients are a public-health concern based on their demonstrated role in the maintenance of bodily functions [5].

Studies evaluating nutrient deficiencies usually focus on children, adolescents, and pregnant or lactating women because these deficiencies are most common among these groups [6,7]. However, to promote health and prevent inadequacies, it is essential to meet individuals’ nutrient requirements for all age groups [8]. Inadequate diets in adults can have negative consequences for individuals and society, contributing to the development of diseases, decreased quality of life, human development deficits, economic productivity loss, and increased health costs [8,9]. In addition, the burden of diseases tends to be higher in the elderly than in other age groups [10]. Therefore, from a public-health point of view, preventing nutritional inadequacies in stages before the onset of the disease is a strategy that can contribute to overcoming this problem.

In Brazil, like in other low- and middle-income countries, obesity and diet-related chronic diseases co-exist with undernutrition, indicating the double burden of malnutrition [11,12]. There is a high prevalence of overweight and obesity but also of critical micronutrient deficiencies affecting many individuals in the country. More than half of the population is overweight [2], while dietary surveys have shown a high prevalence of inadequate intakes of calcium, sodium, magnesium, vitamins A, E, D, and pyridoxine [13].

Furthermore, access to robust dietary intake data is central to nutrition epidemiology and policy applications, but it has been a persistent limitation in nutrition research [14,15]. Major obstacles include the considerable efforts and expenses required in dietary data assessment, especially at a population level, which may lead to data scarcity. Thus, providing information to estimate the burden of disease risk and dietary inadequacies or excesses among the sexes and age subgroups can contribute to understanding the nutritional vulnerabilities of a country and its population [16]. Noteworthily, investigations of dietary intake and inadequacies at the population level provide an assessment of the country’s diet, which may contribute to public policies aimed at improving nutritional status and preventing chronic diseases. Therefore, this study aimed to investigate energy and nutrient intakes and the prevalence of micronutrient inadequacies in the Brazilian adult population and was particularly interested in differences in age and subgroups.

## 2. Materials and Methods

### 2.1. Study Design and Settings

This is a descriptive, cross-sectional study with data from the Brazilian Study of Nutrition and Health (EBANS), a population-based study with a representative sample of individuals aged 15 to 65 years [17] and residents of the urban area of the five macro-regions of Brazil. The EBANS provides data from the Brazilian population to the Latin American Nutrition and Health Study (ELANS) [18], a multicenter study aimed at assessing individuals’ nutritional status, physical activity, and food intake in eight Latin American countries. Data collection occurred between October 2014 and July 2015 in two home visits at least eight days apart.

### 2.2. Subjects and Sample Size

**Figure 1:**
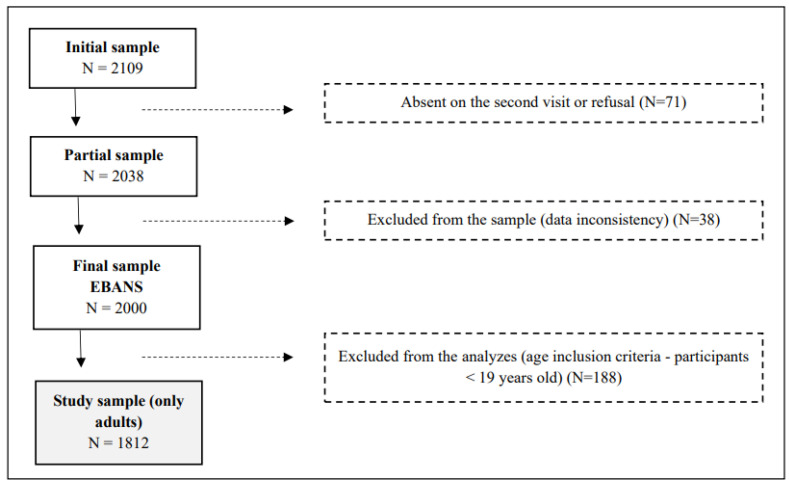
Study flowchart. Adapted from Fisberg et al., 2019 [17].

The cities included in the study were selected by systematic random sampling. The urban conglomerates were chosen systematically, and smaller cities were randomly selected based on population density. Within the conglomerates, the Primary Sampling Units (PSUs), represented by municipalities, districts, and residential areas, and the Secondary Sampling Units (SSUs), defined as the census sectors, were randomly selected. In each SSU, the households were selected systematically, and the interviewer went through each residential block in a clockwise direction, with a sampling interval of three homes. Within the households, the selection of individuals was controlled by quotas, respecting the selection criteria of half the individuals with the closest birthday and the other half with the most distant birthday from the interview date. If selected, participants from the same house could be included in the study sample only if they were from different age strata (e.g., adolescents vs. adults). Because we did not include adolescents in this study, the sample only contained individuals from different households. The overall rationale, design, and study sampling were previously described by Fisberg et al. [17], and the final sample of the EBANS included 2000 participants. The present study focused only on the adult life stage, including data of individuals aged 19 to 65 years, of both sexes, with a final sample size of 1812 respondents. Pregnant and lactating women and people with inborn errors of metabolism were not included. Figure 1 illustrates the sampling flowchart for the study (Figure 1).

### 2.3. Dietary Intake Assessment

Dietary food intake was assessed by two 24-h food recalls (24 HRs) (one at each home visit), representing both weekdays and weekends. The 24 HRs were applied following the standardized procedures by the Multiple Pass Method (MPM) to minimize data-collection errors and with the support of a photo album of portion sizes and utensils to improve the accuracy of the data [19]. Nutritional values were obtained using the Nutrition Data System for Research (NDSR software, 2014 version, NCC, University of Minnesota, Minneapolis, MN, USA), and the agreement was compared with the values of Brazilian food composition tables [20]. Nutritional values in the database were corrected if the concordance rate was not between 80% and 120% with corresponding foods in the Brazilian food composition tables. To do so, the researchers (nutritionists) developed statistical routines to replace the original value with the correct one. Mixed dishes and/or Brazilian preparations not in the NDSR software database were added as recipes, matching the nutrient composition for the Brazilian context. Total daily intake values below 800 kcal/day or above 4000 kcal/day suggested errors in collection or data entry, so the 24 HRs were revised, and the nutritionists made the necessary corrections [21]. The Multiple Source Method (MSM) statistical modeling software [22] was used to estimate the usual intake of nutrients at both population and individual levels.

Macronutrient adequacy was compared to the Acceptable Macronutrient Distribution Range (AMDR), which includes the lower and upper limits of the recommendation for each macronutrient. The AMDR ranges, applicable for both sexes, are between 45 and 65% of the total energy intake from carbohydrates, 20–35% of the total energy intake of total fat, and 10–35% of the total energy intake of protein [23]. Total fiber and saturated fats intakes were evaluated considering the cutoffs proposed by the World Health Organization (WHO) [24,25], which recommends a minimum amount of 25 g per day of total fiber for both men and women and less than 10% of the individual’s total energy intake from saturated fats. For added sugar, we used the Dietary Guidelines for Americans 2020–2025 [26] because WHO guidelines consider recommendations for free sugar but not added sugar [27]. It recommends an intake below 10% of total energy intake from added sugar. To estimate the probability of an inadequate intake of vitamins and minerals, we used the Dietary Reference Intake (DRI) intake targets proposed by the Institute of Medicine (IOM) [23]. The distribution of the usual nutrient intake was compared to the estimated average requirement (EAR), which considers the specific needs of each sex and age group, to obtain the probability of inadequacy for each age range. The EAR represents the average value of the estimated daily intake to meet the needs of half (50%) of the individuals in the population. It is noteworthy that there is no established EAR value for choline. Therefore, we used adequate intake (AI) to evaluate its ingestion. If the individual’s intake is higher than AI, it is most likely that the intake is adequate. We calculated the proportion of individuals with an intake greater than the AI values in the population to determine the probability of adequacy for this nutrient [28].

The participants also reported the use of any type of nutritional supplement, providing the name of the product (when possible) or its main components (e.g., omega 3, vitamin D, or iron, etc.).

### 2.4. Sociodemographic and Anthropometric Measurements

A trained team obtained anthropometric measurements in duplicate during the first home visit following standardized protocols. Body weight and height were measured using a Sanny^®^ portable scale (Sanny^®^, São Paulo, Brazil) with a precision of 0.1 kg and a portable Sanny^®^ stadiometer with a precision of 0.1 cm, respectively. The nutritional status was classified based on the body mass index (BMI), according to the cutoff points established by the WHO for individuals above 19 years [29]. Excess weight included those in the overweight or obesity category (people with BMI ≥ 25 kg/m^2^). The participants self-reported sociodemographic data: age, sex, and educational and socioeconomic levels. The interviewer filled in regional information. Socioeconomic status was classified into three levels, according to the Brazilian Economic Classification Criteria: high (classes A1, A2, and B1), medium (classes B2 and C1), and low (classes C2, D, and E) [30].

We applied the long version of the International Physical Activity Questionnaire (IPAQ) [31] on the second home visit to determine the individual’s physical activity level, using the last seven days as a reference. The long-form IPAQ has been validated internationally to assess total physical activity in individuals from 12 countries with Spearman’s correlation coefficients ranging from 0.46 to 0.96 [32]. Participants were categorized as active (≥150 min/week) or insufficiently active (<150 min/week) using moderate-to-vigorous physical activity guidelines as defined by the WHO [33]. Details on IPAQ data have been published elsewhere [34,35].

### 2.5. Statistical Analysis

Participants’ characteristics were summarized for the general population and stratified by sex and age strata, presented as absolute and relative frequencies. The age-range categories were chosen based on the different life stages of the DRI recommendations [23]. Thus, for comparison among age groups, this study considered younger adults as individuals between 19 and 30 years old; middle-aged adults, between 31 and 50; and older adults, aged 51 to 65. The chi-square test was used to investigate the association between the categorical variables of interest among the different age groups and between sexes. The distribution of continuous variables was checked for normality using the Kolmogorov–Smirnov test to apply the appropriate parametric or non-parametric tests. We applied the Kruskal–Wallis test with Dunn post hoc pairwise multiple comparisons to investigate differences in the distribution of continuous variables. Nutrient intakes and distribution were described by means, medians, standard deviation (SD), and interquartile ranges (IQR). The prevalence of adequate intakes for each nutrient of interest (e.g., macronutrients, fiber, added sugar, and saturated fats) was calculated considering the proportion of individuals within, below, or above recommendations. The probability of inadequacy in micronutrient intakes was calculated using the z-score (using the Z-standard curve) of the mean intake of the population and the EAR of each nutrient, using the equation [36]: Z = (EAR − mean)/SD. All statistical analyses were performed in SPSS software (version 16 for Windows, SPSS Inc., Chicago, IL, USA) with a significance level of 5% (*p*-value < 0.05).

## 3. Results

The study included 1812 adults aged 19–65 (54.3% female). Participants’ characteristics are shown in Table 1. Most of the population was in the middle or low-income strata and had at least a secondary education (high school). There was also a high frequency of people who studied only up to middle school (complete or incomplete), especially among older adults (aged between 51 and 65 years old). In the three age groups, the proportion of women with a low socioeconomic level was higher than men. However, the association among age groups and socioeconomic status was only significant in women (*p* = 0.033) and not in men (*p* = 0.099). Only 8.3% of the general population had a high socioeconomic level, compared to 45.1% and 46.6% in the medium and low levels, respectively (considering all age groups).

The results suggested an association between excess weight (BMI ≥ 25 kg/m²) and the different age groups in the general population and between the sexes (*p* < 0.001). The population had a high prevalence of excess weight, which was even higher with aging. In younger adults (19–30 years), excess weight affected 48% of the population, while in older adults (51–65 years), this prevalence reached 74.5%. This relation between excess weight with age occurred in both sexes, but the excess weight was higher among men than women only for older adults (75.5% vs. 73.9%).

Considering the IPAQ, only 41.3% of the population (considering all age groups together) reported sufficient physical activity, and the proportion of active men was higher than women (46.7% vs. 36.8%). In both sexes, the age group of older adults (51 to 65 years) had the highest percentages of insufficiently active individuals. Still, the association between physical activity and age groups was only significant in women (*p* = 0.010). Only 93 respondents reported using some nutritional supplement, representing just over 5% of the population, with women representing almost twice as many men among those who reported it. However, no association was found between the use of supplements with different age groups.

Table 2 presents the energy and macronutrient-intake distributions across age groups. As expected, men reported a higher energy intake than women in all age groups, but in both sexes, the energy report was lower as the individuals got older (*p* < 0.001). Compared to the AMDR, the mean contribution to the total energy intake (%EI) of all three macronutrients (carbohydrates, proteins, and total fats) was within the recommended range for all age groups. In the present study, a higher proportion of participants met the recommendations for proteins, with over 99% consuming it within the AMDR. For carbohydrates and total fats, the percentages were 84.7% and 80.7%, respectively. However, there is a pattern of more people consuming carbohydrates below the AMDR (13.6%) than above (1.7%), which is the inverse for total fats, with more people consuming them above than below the AMDR (17.6% vs. 1.7%) (Table S1).

Women presented a higher carbohydrate intake than men in all age groups (*p* < 0.001), and a higher total fat (*p* = 0.006) and saturated fat (*p* = 0.003) intake for those in the middle-aged group (31–50 years old). Across age stages, the absolute value of protein consumption decreased with age, with data showing a daily mean intake of 84.3 g, 78.9 g, and 69.9 g, respectively, for young (19–30 years old), middle-aged (31–50 years old), and older adults (51–65 years old). However, older individuals tended to present a higher contribution to %EI from protein than younger and middle-aged adults (*p* < 0.001). Eicosapentaenoic fatty acid (EPA) intake was also higher in older adults compared to younger adults (*p* < 0.001), but DHA intake did not differ among age groups.

Table 3 shows the prevalence of inadequacy in total fiber, added sugar, and saturated fat intake. More than 80% of Brazilian adults had a fiber intake below the recommendation, regardless of age group. This scenario was even worse in women, with the prevalence of inadequacy exceeding 90%. While for women, the prevalence of inadequacy was higher in younger adults, decreasing in the older age group, this relationship was inverse for men, with an increase in inadequacy over age groups. However, these differences were not significant.

There was also a high prevalence of individuals consuming added sugar above the recommended value, and overconsumption was even greater in women compared to men. In both sexes, people aged 19 to 30 years had the highest frequencies of excessive intake of added sugar, while the lowest values were observed among adults aged 51 to 65 (*p* < 0.001). On the other hand, saturated fat intake above the recommendations decreased with age for men (*p* = 0.014), but there were no significant differences among age groups for women (*p* = 0.940). In terms of proportion, women presented a higher prevalence of excess saturated fat intake than men in middle-aged and older-adult age groups.

Table 4 and Table 5 present the distribution and the probabilities of inadequate intakes of vitamins and minerals in the population. The results showed a high probability of inadequacy (above 90%) for vitamins D and E. Vitamin D inadequacy was very similar between the sexes, but vitamin E was more likely to be inadequately consumed among women. Vitamin A also exceeds 53% of inadequacy among men (in all age groups) and 46% among women. Furthermore, the probability of adequacy of choline in the population was very low, reaching 10% only among men in the age group of 19 to 30 years. For the remaining age groups, choline adequacy is around 6%, with the worst-case scenario among older adults.

Thiamine (vitamin B1) showed the best results, with the lowest probabilities of inadequacy in the population. However, some important differences between age groups were noted, such as the significant increase in the inadequacy of thiamine and pyridoxine, especially in men, between young and older adults. While in the first age group, the inadequacy of thiamine did not reach 6%, in older adults, this value was close to 20%. In women, pyridoxine ranged from an inadequacy of 22.1% (among young adults) to 48.4% in older adults.

Regarding minerals, a high probability of inadequacies of calcium and magnesium was found in the population. Except for iron, the probability of inadequacies of the other minerals increased with age. In addition, iron inadequacy in women was higher than in men in all age groups.

## 4. Discussion

This study evaluated energy and nutrient intakes in the Brazilian adult population, investigating differences between sexes and age subgroups. We have also estimated the probability of inadequate intakes of vitamins and minerals in the population. In summary, the results showed many nutrient inadequacies relevant to public health, with a worse scenario among women and older people. There was a high consumption of saturated fat and added sugar and a low adequacy in fiber intake. Additionally, many vitamins and minerals were consumed below the recommendations, resulting in a high probability of inadequacy in the population.

In previous studies, men consumed slightly more calories from protein than women, while women reported more energy from carbohydrates [37]. However, Araujo et al. [38] found a mean energy contribution lower from total fats (about 27%) and higher from carbohydrates (about 55%) than in our research. Our results also showed a decrease in energy intake and calories from total and saturated fats with the advance of age. A possible explanation for this reduction in fat intake with age may be associated with the occurrence and diagnosis of chronic non-communicable diseases, especially cardiovascular diseases, which are more frequent in older people [10]. Usually, with the diagnosis, there are recommendations for changes in lifestyle or the restriction of some food groups, especially fats. This can lead to modifications in the dietary patterns of these individuals, with a reduction in fats, which may also impact energy intake. It may also be related to the decrease in added sugar consumption across age groups because of the diagnosis of type 2 diabetes, which recommends reducing or substituting sugar intake with sweeteners [39]. One factor that might also contribute to these differences in energy intake across ages and between the sexes is the misreporting of energy intake. Older people and women tend to under-report their energy intake more than others [40].

Although the mean percentages of macronutrient contribution to total energy intake were within the AMDR, it is important to mention some limitations, since AMDR considers the distribution of macronutrients within the total caloric intake. Therefore, the distribution may be within the adequate range even when energy intake is inadequate (both above and below). In addition, the AMDR for proteins is quite broad, considering values between 10 and 35% of energy intake to be adequate [23]. However, the health effects of a diet with 10% proteins compared to one with 35% may differ, but still, both would be considered adequate protein intakes by the AMDR.

Most of the population was in the lower categories of education and socioeconomic level, which are relevant in studies that assess nutritional inadequacies and health [41]. These measures are directly associated with the individual’s purchasing power and household food availability, contributing to nutrient intake and adequacy. Especially in lower-income countries, studies show a positive association between the level of education and healthy food choices [42,43].

Most of the population does not meet the requirements for fiber, with worse results for women. Fruits and vegetables, which are important sources of fiber, contribute to a low percentage of the total fiber intake in the population (Table S2), which might justify its low intake. Like in other studies in the Brazilian population, the top three foods that contribute to fiber intake are beans, rice, and bread [44].

EPA and DHA intake varies a lot among studies, and there needs to be a consensus on the recommended dosage for the general population or healthy individuals. However, in our sample, the intake of these two fatty acids was below previous studies. Lu et al. [45] investigated omega-3 fatty acids in the body composition of women with polycystic ovary syndrome and found a mean intake of EPA and DHA of 19.8 mg/day and 18.1 mg/day, respectively. On the other hand, Carballo-Casla et al. [46], investigating fish consumption by Spanish people over 60 years of age, found a much higher mean intake for both fatty acids (EPA—260 mg and DHA—500 mg). Previous studies suggest that doses of 2 to 4 g of EPA/DHA daily can lower triglyceride levels by 25 to 30% [47]. Thus, the Brazilian Cardiology Society suggests that this dose (2–4 g per day) or even higher doses may be recommended only for severe hypertriglyceridemia. However, the actual EPA and DHA intake in the population represents less than 10% of these values. These differences, especially compared to the Spanish context, may be partially attributed to the population’s dietary habits and patterns. Mediterranean countries usually present higher eating frequencies of healthy fats, fish, and omega-3 food sources than Western countries [48]. In Brazil, on the other hand, the dietary intake of polyunsaturated fatty acids is below the recommendations [49]. Major food groups that contribute to its intake, such as fish, the primary food source of EPA and DHA, represent less than 1% of the total energy intake [50]. Evidence that evaluates EPA and DHA intakes is still scarce in Brazil.

We observed a high probability of inadequate intake of vitamins E, D, calcium, and magnesium, with most inadequacies increasing with age. Also, choline adequacy was low in both sexes and even worse in older adults. The most significant difference between the sexes was observed in their iron intake, the micronutrient with the lowest probability of inadequacy in the population. The results are in accordance with previous studies in Brazil and may be related to dietary aspects and public policies in the country. Araujo et al. [38] and Verly Junior et al. [13] also found an inadequate prevalence of over 90% for vitamin D, E, and calcium when evaluating micronutrient intakes in Brazilian adults. The authors also found less than 5% of inadequacy in iron intake in men.

Important calcium food sources, such as milk, yogurt, cheese, fish, and seafood are scarcely consumed by the population [50]. Additionally, the high inadequacy of vitamin D may be explained by the established recommended reference values, considering mainly the US population, assuming little or no sun exposure [51]. This scenario may not be applicable in countries with a predominantly tropical climate, like Brazil, where most of the vitamin D requirement can be met by synthesis from sun exposure. A previous study with a representative sample of São Paulo, the city with the largest population in Brazil, found an inadequate vitamin D intake in nearly 100% of the population. However, serum levels were adequate in almost half of the population [52]. Thus, the EAR for vitamin D in Brazil may be overestimated.

Whole grains are more commonly consumed by women, which may explain the differences between the sexes in inadequacies of B vitamins [53]. In a recent investigation that simulated the effect of replacing refined cereals with whole grains, one of the adverse effects would be the reduction of niacin, riboflavin, pyridoxine, and folate, which could be associated with grain processing [54]. It may also be related to the differences in iron inadequacy between the sexes, which is notably higher in women (in all age groups) than in men. Due to the specific needs associated with the menstrual period, the EAR for iron for adult women is higher than for men, which may increase the magnitude of this difference in the inadequacy of this mineral. After mandatory wheat and corn flour enrichment with iron and folic acid, bread, pasta, and biscuits are the main food sources contributing to these two micronutrients. These are foods commonly restricted in women’s diets due to the perception of their association with weight gain [55].

Nevertheless, this study is not without limitations. Dietary data collection is subject to bias and errors. Although this study assessed food intake using two 24 HRs and adjusted it to estimate the usual intake, especially evaluating micronutrients, this number of collections may not be sufficient to reflect its actual intake. Additionally, only the nutrient intake from food sources was considered in the analysis. However, a robust data collection and analysis methodology was applied to minimize these errors, and the reported supplement use was very low in the population. Furthermore, we had to adopt international cut-off values because the Dietary Guidelines for the Brazilian Population [56] only present qualitative but not quantitative recommendations for evaluating nutrient adequacies. However, even though the cut-offs were not developed specifically for the Brazilian population, these recommendations are based on robust scientific reviews of the current body of evidence commonly related to health and disease risk. Additionally, this study only collected data in the urban areas of the country. However, in 2015, only 14% of the Brazilian population lived in rural areas, according to the World Bank [57]. Thus, a strength of the study is the dietary assessment at the populational level, with a representative sample of Brazilian adults living in urban areas, which can provide information about the nutritional diagnosis for most of the country.

## 5. Conclusions

The present study comprehensively provides information on macro and micronutrient inadequacies and excessive intakes in Brazilian adults. Substantial inadequate nutrient intakes were verified among the population, with those most at-risk being women and older individuals. The results showed a relevant scenario of nutrient inadequacies with public-health concerns, such as fiber, added sugar, vitamin D and E, choline, calcium, and magnesium, which should be addressed through governmental policies, programs, and interventions in the clinical practice of health care professionals. Promoting the consumption of adequate and micronutrient-rich foods, strengthening food fortification, and nutritional supplementation, particularly for groups at risk, are valuable strategies that might help to alleviate these inadequacies, which can improve the population’s nutritional status and, ultimately, the individuals’ quality of life. These findings may contribute to identifying subgroups in the population that are vulnerable to inadequate nutrient intake, to target better and monitor public health policies, and to assist in reducing nutritional problems in the population.

## Figures and Tables

**Table 1:** Sociodemographic, nutritional status, physical activity level, and supplement use of Brazilian adults in the EBANS study, stratified by sex and age group. Brazil, 2015.

	Total Population (N = 1812)							Male (N = 828)							**Female (N = 984)**						
	Age Strata (Years)							Age Strata (Years)							**Age Strata (Years)**						
	19–30		31–50		51–65			19–30		31–50		51–65			**19–30**		**31–50**		**51–65**		
	N	%	N	%	N	%	*p*-Value	N	%	N	%	N	%	*p*-Value	**N**	**%**	**N**	**%**	**N**	**%**	***p*** **-Value**
	573	32.0	851	47.0	388	21.0		295	16.0	394	22.0	139	8.0		**278**	**15.0**	**457**	**25.0**	**249**	**14.0**	
**Education level**																					
None to middle school	196	23.8	380	46.2	247	30.0	**<0.001**	95	32.2	178	45.2	86	61.9	**<0.001**	101	36.3	202	44.2	161	64.7	**<0.001**
High school	345	60.2	372	43.7	104	26.8		185	62.7	172	43.7	38	27.3		160	57.6	200	43.8	66	26.5	
College/university degree	32	5.6	99	11.6	37	9.5		15	5.1	44	11.2	15	10.8		17	6.1	55	12.0	22	8.8	
**Socioeconomic status**																					
High	44	7.7	78	9.2	31	8.0	**0.003**	26	8.8	33	8.4	13	9.4	0.099	18	6.5	45	9.9	18	7.2	**0.033**
Middle	269	47.0	402	47.2	143	36.9		157	53.2	200	50.8	55	39.6		112	40.3	202	44.2	88	35.3	
Low	260	45.4	371	43.6	214	55.2		112	38.0	161	40.9	71	51.1		148	53.2	210	46.0	143	57.4	
**Excess weight**																					
BMI < 25 kg/m^2^	298	52.0	303	35.6	99	25.5	**<0.001**	156	52.9	148	37.6	34	24.5	**<0.001**	142	51.1	155	33.9	65	26.1	**<0.001**
BMI ≥ 25 kg/m^2^	275	48.0	548	64.4	289	74.5		139	47.1	246	62.4	105	75.5		136	48.9	302	66.1	184	73.9	
**PAL (IPAQ)**																					
Insufficiently active	300	54.4	462	56.6	233	62.1	0.058	131	46.1	210	54.8	75	55.2	0.058	169	63.1	252	58.1	158	66.1	**0.010**
Active	252	45.7	355	43.5	142	37.9		153	53.9	173	45.2	61	44.9		99	36.9	182	41.9	81	33.9	
**Supplement use**																					
No	488	85.2	722	84.8	318	82.0	0.624	257	87.1	345	87.6	115	82.7	0.558	231	83.1	377	82.5	203	81.5	0.986
Yes	26	4.5	42	4.9	25	6.4		11	3.7	14	3.6	9	6.5		15	5.4	28	6.1	16	6.4	
Not informed	59	10.3	87	10.2	45	11.6		27	9.2	35	8.9	15	10.8		32	11.5	52	11.4	30	12.1	

*p*-value: Chi-square test. BMI: Body Mass Index. PAL: Physical-Activity Level. IPAQ: International Physical Activity Questionnaire.

**Table 2:** Energy intake and macronutrient distributions reported by Brazilian adults in the EBANS study, stratified by sex and age group. Brazil, 2015.

Nutrient Intake	Total Population (n = 1812)					K–Wallis	Male (n = 828)					Female (n = 984)					K–Wallis
	Mean	SD	P25	P50	P75	*p*-Value *	Mean	SD	P25	P50	P75	Mean	SD	P25	P50	P75	*p* Value **
**Energy (kcal)**																	
19–30 years	1978	623	1564	1888	2275	**<0.001 a,b,c**	2252	651	1824	2162	2523	1687	430	1366	1691	1921	**<0.001 e,f,h,i,j,k,l**
31–50 years	1830	576	1417	1753	2151		2088	594	1690	2034	2416	1609	458	1278	1564	1866	
51–65 years	1566	464	1230	1511	1844		1827	474	1493	1810	2077	1420	389	1147	1341	1648	
**Carbohydrates (% EI)**																	
19–30 years	50.78	6.65	46.76	51.16	55.05	0.103	49.89	6.71	45.19	49.94	54.54	51.72	6.46	47.98	52.01	55.91	**<0.001 j,k,l**
31–50 years	50.13	6.61	45.76	50.29	54.71		49.30	6.76	44.81	49.62	53.67	50.84	6.4	46.59	50.67	55.33	
51–65 years	50.35	7.25	46.06	50.81	54.79		48.64	6.82	44.27	48.96	53.21	51.30	7.31	46.98	51.64	56.14	
**Proteins (% EI)**																	
19–30 years	17.26	3.31	15.09	16.97	19.15	**0.001 b,c**	17.58	3.42	15.38	17.11	19.6	16.92	3.15	14.9	16.77	18.7	**<0.001 e,f**
31–50 years	17.53	3.37	15.18	17.21	19.46		17.51	3.33	15.35	17.21	19.58	17.54	3.41	15.11	17.21	19.32	
51–65 years	18.11	3.54	15.72	17.81	19.92		18.64	3.57	16.37	18.61	20.28	17.82	3.5	15.45	17.31	19.69	
**Total fats (% EI)**																	
19–30 years	30.18	4.91	27.32	30.08	33.37	0.383	29.89	4.78	27.22	30.02	33.22	30.48	5.03	27.37	30.38	33.6	**0.006 k**
31–50 years	29.8	5.25	26.74	30.05	33.12		28.99	5.46	25.92	29.47	32.68	30.5	4.96	27.46	30.45	33.81	
51–65 years	29.7	5.38	26.43	29.83	33.31		29.3	5.25	25.94	29.71	32.88	29.93	5.46	26.7	30.25	33.54	
**Saturated fats (% EI)**																	
19–30 years	9.95	1.99	8.69	9.9	11.33	0.274	9.85	1.9	8.62	9.93	11.25	10.06	2.07	8.75	9.87	11.55	**0.003 k**
31–50 years	9.84	2.13	8.47	9.7	11.23		9.57	2.22	8.16	9.47	10.91	10.08	2.03	8.73	9.9	11.39	
51–65 years	9.81	2.17	8.35	9.8	11.1		9.58	2.05	8.25	9.58	10.93	9.93	2.23	8.53	9.94	11.27	
**Unsaturated fats (% EI)**																	
19–30 years	7.61	1.87	6.27	7.52	8.76	0.319	7.46	1.82	6.19	7.5	8.51	7.77	1.92	6.47	7.57	8.94	0.093
31–50 years	7.45	1.86	6.31	7.39	8.47		7.26	1.82	6.13	7.33	8.36	7.61	1.87	6.47	7.4	8.68	
51–65 years	7.41	1.79	6.26	7.31	8.43		7.31	1.65	6.19	7.39	8.26	7.47	1.86	6.27	7.31	8.62	
**EPA (mg)**																	
19–30 years	11.81	8.42	7.51	10.5	13.32	**<0.001 a,b**	10.98	7.19	7.18	9.56	13.02	12.68	9.49	8.58	11.27	13.64	**<0.001 e,j,k**
31–50 years	12.8	8.22	8.34	11.08	14.57		12.19	8.51	7.9	10.31	13.75	13.32	7.94	9.21	11.74	15.27	
51–65 years	13.49	10.73	8.55	11.58	14.67		12.93	8.31	8.47	11.06	14.7	13.8	11.87	8.96	11.92	14.67	
**DHA (mg)**																	
19–30 years	48.16	33.58	29.41	40.23	54.51	0.187	49.46	37.06	29.41	40.69	55.86	46.79	29.43	29.28	39.19	54.1	0.550
31–50 years	51.05	36.38	29.81	40.93	58.58		51.72	36.33	30.02	41.1	58.63	50.48	36.46	29.71	40.62	57.73	
51–65 years	50.4	39.84	28.28	39.26	57.41		52.03	35.75	26.2	38.68	64.18	49.49	42	29.25	39.31	53.35	

SD: Standard deviation. % EI: Nutrient intake as a percentage of total energy intake. EPA: Eicosapentaenoic fatty acid. DHA: Docosahexaenoic fatty acid. * Kruskal–Wallis test with post hoc pairwise comparisons: analysis to evaluate the differences among age groups in the overall population: (a) significant differences between age groups 19–30 years and 31–50 years; (b) significant differences between age groups 19–30 years and 51–65 years; (c) significant differences between age groups 31–50 years and 51–65 years. ** Kruskal–Wallis test with post hoc pairwise comparisons: analysis to evaluate the differences between sexes and the three age groups: (d) significant differences between age groups 19–30 years and 31–50 years among men; (e) significant differences between age groups 19–30 years and 51–65 years among men; (f) significant differences between age groups 31–50 years and 51–65 years among men; (g) significant differences between age groups 19–30 years and 31–50 years among women; (h) significant differences between age groups 19–30 years and 51–65 years among women; (i) significant differences between age groups 31–50 years and 51–65 years among women; (j) significant differences between 19 to 30 years (male) vs. 19 to 30 years (female); (k) significant differences between 31 and 50 years (male) vs. 31 and 50 years (female); (l) significant differences between 51 and 65 years (male) vs. 51 and 65 years (female).

**Table 3:** Prevalence of inadequacy in total fiber, added sugar, and saturated fat intake in Brazilian adults, stratified by sex and age group. Brazil, 2015.

Nutrient	Total Population (n = 1812)			Male (n = 828)			Female (n = 984)		
	n	%	*p*-Value	n	%	*p*-Value	n	%	*p*-Value
**Total fiber (g)**	**<25 g/day**			**<25 g/day**			**<25 g/day**		
**Total—19–65 years**	1508	83.22		580	70.05		928	94.31	
19–30 years	463	80.8	0.062	197	66.78	0.170	266	95.68	0.354
31–50 years	709	83.31		278	70.56		431	94.31	
51–65 years	336	86.6		105	75.54		231	92.77	
**Added sugar (% EI)**	**>10% of EI**			**>10% of EI**			**>10% of EI**		
**Total—19–65 years**	1121	61.87		453	54.71		668	67.89	
19–30 years	404	70.51	*p* < 0.001	184	62.37	*p* < 0.001	220	79.14	*p* < 0.001
31–50 years	523	61.46		212	53.81		311	68.05	
51–65 years	194	50		57	41.01		137	55.02	
**Saturated fat (%EI)**	**>10% of EI**			**>10% of EI**			**>10% of EI**		
**Total—19–65 years**	814	44.92		346	41.79		468	47.56	
19–30 years	273	47.64	0.225	143	48.47	0.014	130	46.76	0.940
31–50 years	366	43.01		148	37.56		218	47.7	
51–65 years	175	45.1		55	39.57		120	48.19	

*p*-value: Chi-square test. % EI: Nutrient intake as a percentage of total energy intake.

**Table 4:** Distribution and probabilities of inadequate vitamin intakes in Brazilian adults, stratified by sex and age group. Brazil, 2015.

Micronutrient	Brazil (n = 1812)		Male (n = 828)				Female (n = 984)			
	Mean	SD	Mean	SD	EAR	Inadequacy (%)	Mean	SD	EAR	Inadequacy (%)
**Vitamin A (mcg)**										
19–30 years	554.51	590.02	567.77	706.99	625	53.19%	540.43	433.56	500	46.41%
31–50 years	547.55	459.15	557.57	497.32	625	55.57%	538.91	423.85	500	46.41%
51–65 years	527.48	461.74	560.79	503.24	625	55.17%	508.88	436.81	500	49.20%
**Vitamin C (mg)**										
19–30 years	90.52	84.76	90.04	90.16	75	43.25%	91.03	78.80	60	34.83%
31–50 years	91.56	79.72	90.19	84.13	75	42.86%	92.74	75.78	60	33.36%
51–65 years	98.00	83.26	94.88	82.79	75	40.52%	99.74	83.63	60	31.56%
**Vitamin D (mcg)**										
19–30 years	3.54	2.16	3.79	2.27	10	99.69%	3.28	2.00	10	99.88%
31–50 years	3.32	1.85	3.52	1.96	10	99.95%	3.16	1.73	10	99.87%
51–65 years	3.12	1.73	3.24	1.72	10	99.99%	3.05	1.73	10	99.46%
**Vitamin E (mg)**										
19–30 years	7.40	2.73	8.03	2.86	12	91.77%	6.72	2.43	12	98.50%
31–50 years	7.06	2.91	7.74	3.22	12	90.66%	6.47	2.48	12	98.71%
51–65 years	6.55	2.61	7.41	3.09	12	93.06%	6.07	2.15	12	99.70%
**Thiamin (mg)**										
19–30 years	1.62	0.51	1.82	0.52	1.0	5.82%	1.42	0.42	0.9	10.93%
31–50 years	1.60	0.57	1.78	0.58	1.0	9.01%	1.45	0.52	0.9	14.23%
51–65 years	1.50	0.66	1.76	0.90	1.0	19.77%	1.36	0.41	0.9	13.14%
**Riboflavin (mg)**										
19–30 years	1.38	0.47	1.52	0.50	1.1	19.77%	1.23	0.39	0.9	20.05%
31–50 years	1.30	0.45	1.43	0.47	1.1	24.20%	1.18	0.39	0.9	23.27%
51–65 years	1.17	0.37	1.33	0.41	1.1	28.77%	1.08	0.32	0.9	28.43%
**Pyridoxine (mg)**										
19–30 years	1.74	0.72	2.01	0.80	1.1	12.17%	1.46	0.47	1.1	22.06%
31–50 years	1.67	0.70	1.95	0.78	1.1	13.79%	1.43	0.50	1.1	25.14%
51–65 years	1.49	0.65	1.80	0.82	1.4	31.21%	1.32	0.45	1.3	48.40%
**Vitamin B12 (mcg)**										
19–30 years	4.41	2.38	4.97	2.57	2.0	12.30%	3.82	2.01	2.0	18.14%
31–50 years	4.26	2.34	4.76	2.58	2.0	14.23%	3.83	2.01	2.0	18.14%
51–65 years	3.87	1.63	4.71	1.95	2.0	8.23%	3.41	1.19	2.0	11.70%
Choline *						**Prob. Adeq. (%)**				**Prob. Adeq. (%)**
19–30 years	337.85	123.23	387.9	132.47	550 *	10.51%	284.74	85.042	425 *	6.12%
31–50 years	325.45	113.15	370.34	118.32	550 *	6.60%	286.75	92.497	425 *	6.78%
51–65 years	295.85	98.167	351.12	107.99	550 *	2.28%	265	76.691	425 *	4.02%

SD: Standard deviation. EAR: Estimated Average Requirement. Inadequacy (%) expresses the probability of inadequacy based on EAR values for each micronutrient. * AI—Adequate intake. For choline, the probability of adequacy is estimated based on AI values (EAR is not available).

**Table 5:** Distribution and probabilities of inadequate mineral intakes in Brazilian adults, stratified by sex and age group. Brazil, 2015.

Micronutrient	Brazil (n = 1812)		Male (n = 828)				Female (n = 984)			
	Mean	SD	Mean	SD	EAR	Inadequacy (%)	Mean	SD	EAR	Inadequacy (%)
**Calcium (mg)**										
19–30 years	470.80	244.02	509.13	265.31	800	86.42%	430.12	212.21	800	95.91%
31–50 years	451.66	239.22	463.56	219.30	800	93.70%	441.40	254.94	800	92.07%
51–65 years	424.57	204.40	454.31	213.32	800	95.73%	407.98	197.75	1000	99.86%
**Iron (mg)**										
19–30 years	12.36	4.18	14.20	4.18	6.0	2.50%	10.40	3.17	8.10	23.27%
31–50 years	11.36	4.11	13.22	4.38	6.0	4.95%	9.75	3.05	8.10	29.46%
51–65 years	9.98	3.27	11.77	3.02	6.0	2.81%	8.98	2.97	5.00	9.01%
**Magnesium (mg)**										
19–30 years	216.45	71.79	246.95	77.02	330	85.99%	184.09	48.01	255	93.06%
31–50 years	211.69	69.51	240.75	73.45	350	93.19%	186.64	54.77	265	92.36%
51–65 years	199.76	62.56	228.55	65.74	350	96.78%	183.69	54.58	265	93.19%
**Zinc (mg)**										
19–30 years	12.40	5.33	14.53	6.04	9.4	19.77%	10.13	3.15	6.8	14.46%
31–50 years	11.51	4.48	13.29	4.87	9.4	21.19%	9.97	3.43	6.8	17.88%
51–65 years	10.41	4.05	12.29	3.95	9.4	23.27%	9.36	3.71	6.8	24.51%

SD: Standard deviation. EAR: Estimated Average Requirement. Inadequacy (%) expresses the probability of inadequacy based on EAR values for each micronutrient.

## Data Availability

Data is available upon reasonable request. It is not publicly available due to confidentiality/ethical restrictions. Data can be available through the EBANS institutional board for researchers who meet the criteria for access to confidential data. For data requests, please contact: Instituto PENSI (PENSI Institute) Ave. Angelica 2071, 2nd floor. Sao Paulo, Brazil. 01221-200. Phone: +55-11-2526-2525. Email: pesqeasy@pensi.org.br.

## Supplementary Materials

The following supporting information can be downloaded at: https://www.mdpi.com/article/10.3390/nu16050750/s1, Table S1: Macronutrient intake in adults in the urban areas of Brazil, 2015.; Table S2: Top 10 food sources contributing to nutrient total intake in adults in the urban areas of Brazil, 2015.
