# Supplementary material for: Exploring Diet and Nutrient Insufficiencies across Age Groups: Insights from a Population-Based Study of Brazilian Adults
Source: Nutrients. 2024 Mar 5;16(5):750. doi: 10.3390/nu16050750 (PMC10934561; doi:10.3390/nu16050750)
Supplement: Supplementary file 1 [file nutrients-16-00750-s001.zip › Table S1.pdf]

Table S1: Macronutrient intake in adults in the urban areas of Brazil, 2015.

	Carbohydrates	Proteins	Total fats
**AMDR**	**45-65%**	**10-35%**	**20-35%**
	**n (%)**	**n (%)**	**n (%)**
**Total population (n = 1812)**			
Below AMDR	247 (13.6%)	6 (0.3%)	30 (1.7%)
Within AMDR	1535 (84.7%)	1806 (99.7%)	1463 (80.7%)
Above AMDR	30 (1.7%)	0 (0.00%)	319 (17.6%)
**Male (n = 828)**			
Below AMDR	121 (14.6%)	3 (0.4%)	19 (2.3%)
Within AMDR	697 (84.2%)	825 (99.6%)	675 (81.5%)
Above AMDR	10 (1.2%)	0 (0.00%)	134 (16.2%)
**Female (n = 984)**			
Below AMDR	126 (12.8%)	3 (0.3%)	11 (1.1%)
Within AMDR	838 (85.2%)	981 (99.7%)	788 (80.1%)
Above AMDR	20 (2.0%)	0 (0.00%)	185 (18.8%)

Acceptable macronutrient distribution range (AMDR)
