# Supplementary material for: Exploring Diet and Nutrient Insufficiencies across Age Groups: Insights from a Population-Based Study of Brazilian Adults
Source: Nutrients. 2024 Mar 5;16(5):750. doi: 10.3390/nu16050750 (PMC10934561; doi:10.3390/nu16050750)
Supplement: Supplementary file 1 [file nutrients-16-00750-s001.zip › Table S2.pdf]

Table S2: Top 10 food sources contributing to nutrient total intake in adults in the urban areas of Brazil, 2015.

		PROTEIN			
**FoodID**	**Food name (category)**	**Contribution to total intake (%)**	**Frequency of intake (n)**	**Mean**	**SD**
6510	beef, steak - beef, sirloin, visible fat eaten	11.92	479	132.1	85.1
6256	poultry, chicken, breast, skin removed before cooking	7.38	346	97.9	78.5
8741	bread, loaf, French bread	7.34	1331	66.6	32.6
6356	beef, cubes or stew meat, tip or kabobs, visible fat eaten	6.15	299	95.1	70.9
11009	vegetables, beans, brown, canned - drained, regular	5.45	1426	55.7	41.2
9097	fish and seafood, cod, cooked from fresh or frozen, Atlantic	5.06	152	206.7	138.4
4338	milk, whole (3.5 - 4% fat)	3.63	1142	143.0	117.4
18389	beef, hamburger or ground beef, 10% fat (90% lean meat)	3.61	292	66.0	67.0
8558	grains, rice, white, regular cooking, cooked in salted water	3.55	1820	102.9	67.3
6271	poultry, chicken, thigh, skin eaten	3.19	231	69.5	55.3
		**OMEGA 3**			
**FoodID**	**Food name (category)**	**Contribution to total intake (%)**	**Frequency of intake (n)**	**Mean**	**SD**
9097	fish and seafood, cod, cooked from fresh or frozen, Atlantic	41.03	152	206.7	138.4
114107	fish and seafood, salmon, cooked from fresh or frozen, coho (silver), farmed	10.34	7	200.0	28.9
6271	poultry, chicken, thigh, skin eaten	9.52	231	69.5	55.3
6256	poultry, chicken, breast, skin removed before cooking	8.64	346	97.9	78.5
1798	ingredient, egg, whole	7.10	499	44.0	43.0
9459	fish and seafood, sardines, canned in oil, drained	5.69	19	69.4	51.4
9462	fish and seafood, sardines, cooked from fresh or frozen	4.98	7	69.9	43.9
6255	poultry, chicken, breast, skin eaten	1.36	79	101.4	77.9
9162	fish and seafood, hake, cooked from fresh or frozen, silver	0.89	1	120.0	.
975	eggs, boiled	0.82	87	29.6	27.6
		**IRON**			
**FoodID**	**Food name (category)**	**Contribution to total intake (%)**	**Frequency of intake (n)**	**Mean**	**SD**
11009	vegetables, beans, brown, canned - drained, regular	20.02	1426	55.7	41.2
8741	bread, loaf, French bread	18.03	1331	66.6	32.6
6510	beef, steak - beef, sirloin, visible fat eaten	5.21	479	132.1	85.1
11014	vegetables, beans, black, canned - drained, regular	3.12	218	49.2	26.1
8531	grains, pasta or noodles, spaghetti noodles, white, cooked in salted water	3.11	343	124.6	95.0
1821	ingredient, flour, white all-purpose, enriched	2.86	616	22.8	26.1
18389	beef, hamburger or ground beef, 10% fat (90% lean meat)	2.80	292	66.0	67.0
6356	beef, cubes or stew meat, tip or kabobs, visible fat eaten	2.34	299	95.1	70.9
27320	crackers, wheat, reduced fat	1.89	257	36.1	31.8
6395	beef, organ meats, liver	1.81	35	184.3	92.7
		**VITAMIN D**			
**FoodID**	**Food name (category)**	**Contribution to total intake (%)**	**Frequency of intake (n)**	**Mean**	**SD**
4338	milk, whole (3.5 - 4% fat)	33.96	1142	143.0	117.4
1798	ingredient, egg, whole	7.79	499	44.0	43.0
6510	beef, steak - beef, sirloin, visible fat eaten	7.22	479	132.1	85.1
9097	fish and seafood, cod, cooked from fresh or frozen, Atlantic	5.89	152	206.7	138.4
4350	milk, unprepared dry powder, whole	5.40	206	15.3	13.6
114107	fish and seafood, salmon, cooked from fresh or frozen, coho (silver), farmed	3.69	7	200.0	28.9

FoodID	Food name (category)	Contribution to total intake (%)	Frequency of intake (n)	Mean	SD
25488	milk, mixtures and milk drinks, cocoa or hot chocolate, dry mix - unprepared, regular, chocolate flavors	3.09	159	21.6	11.7
4783	yogurt, plain, whole milk (3-4% fat)	2.75	39	216.3	129.4
3203	cheese, American cheese, process	2.19	44	40.6	44.5
4787	yogurt, fruited, regular, whole milk (3-4% fat)	2.01	35	175.9	73.1
		**TOTAL FIBER**			
**FoodID**	**Food name (category)**	**Contribution to total intake (%)**	**Frequency of intake (n)**	**Mean**	**SD**
11009	vegetables, beans, brown, canned - drained, regular	39.33	1426	55.7	41.2
8558	grains, rice, white, regular cooking, cooked in salted water	8.73	1820	102.9	67.3
8741	bread, loaf, French bread	6.19	1331	66.6	32.6
11014	vegetables, beans, black, canned - drained, regular	5.25	218	49.2	26.1
7581	vegetables, potato, boiled, without skin	2.29	456	117.5	137.9
8485	grains, pasta or noodles, egg noodles, white, cooked in salted water	2.08	86	148.0	96.9
3159	vegetables, tomato, raw	1.78	590	86.5	169.1
4974	fruit, banana, fresh or ripe	1.77	423	71.8	50.6
11137	fruit, guava (guayaba), fresh common	1.56	68	140.5	119.1
5217	fruit, orange, fresh	1.30	148	167.5	135.0
		**CALCIUM**			
**FoodID**	**Food name (category)**	**Contribution to total intake (%)**	**Frequency of intake (n)**	**Mean**	**SD**
4338	milk, whole (3.5 - 4% fat)	24.57	1142	143.0	117.4
3264	cheese, Mozzarella cheese, whole milk	11.40	339	31.4	28.2
11009	vegetables, beans, brown, canned - drained, regular	5.25	1426	55.7	41.2
4350	milk, unprepared dry powder, whole	3.43	206	15.3	13.6
4864	cheese, American cheese, process - reduced fat	2.83	76	52.5	38.7
3265	cheese, Mozzarella cheese, part skim milk, regular	2.44	80	26.5	27.9
10784	ingredient, cornmeal - dry, yellow (degermed, enriched)	1.86	123	56.5	56.5
8741	bread, loaf, French bread	1.73	1331	66.6	32.6
4341	milk, skim, nonfat or fat free	1.70	65	175.4	169.8
149	beverages, beer, regular	1.67	206	1655.7	1599.0
		**VITAMIN A**			
**FoodID**	**Food name (category)**	**Contribution to total intake (%)**	**Frequency of intake (n)**	**Mean**	**SD**
6395	beef, organ meats, liver	56.89	35	184.3	92.7
10719	fats, margarine, regular, tub, salted, soybean oil	6.54	1007	14.7	17.6
4338	milk, whole (3.5 - 4% fat)	4.51	1142	143.0	117.4
7647	vegetables, sweet potato, boiled	3.06	22	291.6	237.2
1058	fats, butter, regular, salted	2.93	333	15.5	14.8
25488	milk, mixtures and milk drinks, cocoa or hot chocolate, dry mix - unprepared, regular, chocolate flavors	1.66	159	21.6	11.7
2964	vegetables, carrots, cooked from fresh	1.63	212	39.6	36.7
4864	cheese, American cheese, process - reduced fat	1.52	76	52.5	38.7
1798	ingredient, egg, whole	1.52	499	44.0	43.0
13930	vegetables, lettuce, green leaf	1.51	476	34.9	25.8
		**VITAMIN C**			
**FoodID**	**Food name (category)**	**Contribution to total intake (%)**	**Frequency of intake (n)**	**Mean**	**SD**
8064	beverages, juice or flavored drink, orange, juice, fresh	18.97	150	284.0	143.0
107891	beverages, juice or flavored drink, acerola juice	9.42	46	106.3	63.3
5217	fruit, orange, fresh	8.01	148	167.5	135.0
3159	vegetables, tomato, raw	6.57	590	86.5	169.1
21192	beverages, juice or flavored drink, dry mix - unprepared, fruit flavored drink, presweetened, with sugar	5.95	451	16.0	32.6

7942	beverages, juice or flavored drink, black currant juice	5.64	15	331.1	82.1
5221	fruit, papaya, fresh	5.36	82	130.8	98.4
11137	fruit, guava (guayaba), fresh common	4.68	68	140.5	119.1
8066	beverages, juice or flavored drink, orange, juice, purchased ready-to-drink, not fortified	3.02	207	71.6	145.2
5182	fruit, mango, fresh	3.02	135	121.7	93.5
		**VITAMIN E**			
**FoodID**	**Food name (category)**	**Contribution to total intake (%)**	**Frequency of intake (n)**	**Mean**	**SD**
1177	fats, oil, soybean - unhydrogenated	18.15	6215	3.1	4.1
10719	fats, margarine, regular, tub, salted, soybean oil	11.26	1007	14.7	17.6
11009	vegetables, beans, brown, canned - drained, regular	9.53	1426	55.7	41.2
11709	fruit, passion fruit (maracuya) - fresh	4.47	77	37.9	17.6
9097	fish and seafood, cod, cooked from fresh or frozen, Atlantic	3.84	152	206.7	138.4
107857	beverages, tea, Yerba Mate	3.66	37	576.6	541.9
1169	fats, oil, olive	3.39	541	4.4	5.4
6510	beef, steak - beef, sirloin, visible fat eaten	2.19	479	132.1	85.1
3159	vegetables, tomato, raw	2.18	590	86.5	169.1
6381	beef, jerky, regular	2.15	83	78.1	75.5
		**MAGNESIUM**			
**FoodID**	**Food name (category)**	**Contribution to total intake (%)**	**Frequency of intake (n)**	**Mean**	**SD**
11009	vegetables, beans, brown, canned - drained, regular	17.61	1426	55.7	41.2
7772	beverages, coffee, regular (caffeinated), made from ground	6.80	1894	136.1	84.6
149	beverages, beer, regular	6.30	206	1655.7	1599.0
8741	bread, loaf, French bread	5.84	1331	66.6	32.6
4338	milk, whole (3.5 - 4% fat)	4.31	1142	143.0	117.4
4974	fruit, banana, fresh or ripe	3.05	423	71.8	50.6
8558	grains, rice, white, regular cooking, cooked in salted water	2.97	1820	102.9	67.3
6510	beef, steak - beef, sirloin, visible fat eaten	2.34	479	132.1	85.1
11014	vegetables, beans, black, canned - drained, regular	2.26	218	49.2	26.1
8531	grains, pasta or noodles, spaghetti noodles, white, cooked in salted water	2.03	343	124.6	95.0
